# A Five-Gene-Based Prognostic Signature for Hepatocellular Carcinoma

**DOI:** 10.3389/fmed.2021.681388

**Published:** 2021-09-08

**Authors:** Dazhi Tian, Yang Yu, Li Zhang, Jisan Sun, Wentao Jiang

**Affiliations:** Department of Liver Transplantation, Tianjin First Central Hospital, Tianjin, China

**Keywords:** hepatocellular carcinoma, CDC20, TOP2A, RRM2, UBE2C, AOX1, immune cells, multi-databases-based joint analysis

## Abstract

**Objective:** This study intends to identify potential prognostic marker genes associated with the prognosis of patients suffering from hepatocellular carcinoma (HCC) based on TCGA and GEO analysis.

**Methods:** TCGA-LIHC cohort was downloaded and the data related to HCC were extracted from The Cancer Genome Atlas (TCGA) database and subjected to differential analysis. HCC-related gene expression datasets were retrieved from the GEO database, followed by differential analysis. After intersection of the results of TCGA and GEO databases, gene interaction analysis was performed to obtain the core genes. To identify the genes related to the prognosis of HCC patients, we conducted univariate and multivariate Cox analyses.

**Results:** Based on differential analysis of TCGA database, 854 genes were differentially expressed in HCC, any of which might link to the occurrence and progression of HCC. Meanwhile, joint analysis of HCC-related gene expression datasets in the GEO database screened 214 genes. Five core genes CDC20, TOP2A, RRM2, UBE2C and AOX1 were significantly associated with the prognosis of HCC patients and the risk model based on these five genes effectively predicted the prognosis of HCC patients.

**Conclusion:** Collectively, our data suggest that CDC20, TOP2A, RRM2, UBE2C and AOX1 may be the key genes affecting the prognosis of patients with HCC. The five-gene signature could accurately predict the prognosis of HCC patients.

## Introduction

Hepatocellular carcinoma (HCC) is identified as the most frequently occurring primary liver cancer as well as one of the leading reasons for cancer-related death on a global scale (1). It is known that cirrhosis of the liver is a crucial risk factor for the development of HCC, and this group of patients thus need surveillance for early detection, which is best obtained by measuring alpha-fetoprotein levels and regular ultrasound of the liver (2). The first-line agents for treatment of advanced HCC include sorafenib, combined use of atezolizumab and bevacizumab, and lenvatinib, and adoptive cell transfer therapy, oncolytic viruses, in addition to locoregional therapies also have a potential benefit (3). Of note, the efficacy of optimal treatment for HCC has been limited by heterogeneity of this malignancy, which has constrained the advancement in personalized therapy (4).

Cell division cycle 20 (CDC20), in combination with G2 and S-phase expressed 1 (GTSE1), proliferating cell nuclear antigen (PCNA), and minichromosome maintenance complex component 6 (MCM6), could have effects on cell cycle of HCC, while serving as markers for poor prognosis (5). Moreover, CDC20 was identified as an independent prognostic factor in HCC and is able to promote HCC invasion and migration (6). According to a previous study, topoisomerase II alpha (TOP2A) and ribonucleotide reductase subunit M2 (RRM2) were upregulated hub genes in HCC, and shared an association with lower survival rate of patients with HCC (7). Strikingly, ubiquitin-conjugating enzyme 2C (UBE2C) was also found to be one of the key genes in HCC (8). HCC samples had high expression of UBE2C than adjacent normal biopsies (9). Notably, a diminished or completely absent expression of alcohol oxidase 1 (AOX1) was observed in HCC (10). In the current study, we performed a joint analysis in multi-databases to explore prognostic marker genes related to the prognosis of patients with HCC.

## Materials and Methods

### Ethics Statement

The current study was ratified by the Ethics Committee of Tianjin First Central Hospital and performed in strict accordance with the *Declaration of Helsinki*. All participants signed informed consent documentation before enrollment.

### Data Collection and Differential Analysis

The Cancer Genome Atlas (TCGA) liver hepatocellular carcinoma (TCGA-LIHC) cohort was downloaded from the UCSC Xena database (https://xena.ucsc.edu/). In TCGA, 50 normal liver tissue samples and 374 HCC tissue samples were downloaded, and the data type was RNA-seq. Three HCC-related gene expression datasets, GSE45267, GSE49515 and GSE89377, were retrieved from the Gene Expression Omnibus (GEO) database (https://www.ncbi.nlm.nih.gov/gds), followed by extraction of the HCC samples and corresponding normal tissue samples (Table 1). The annotation platform of the GSE45267 and GSE49515 datasets was GPL570, and that of the GSE89377 dataset was GPL6947. The expression data obtained from TCGA database were subjected to differential analysis using the “edgeR” package (11). The three datasets obtained from the GEO database were subjected to batch correction using the R language “SVA” package (12) and differential analysis of the obtained data was then conducted using the R language “limma” package. The false discovery rate (FDR)-adjusted *p* value < 0.05 and |log fold change (FC)| > 1 were set as the threshold to screen the differentially expressed genes. A heat map of the expression of differentially expressed genes was plotted by the “pheatmap” package. Gene Ontology (GO) and Kyoto Encyclopedia of Genes and Genomes (KEGG) enrichment analysis of specified genes was performed using the R “clusterProfiler” package (13). GO entry identifiers were composed of biological process, cellular component and molecular function, and those findings with adjusted *p* value < 0.05 were considered significantly enriched.

**Table 1:** Collection of expression data of HCC.

**Data number**	**Sample sources**	**Number of normal samples**	**Number of tumor samples**
TCGA	TCGA database	50	374
GSE45267	GEO database	17	15
GSE49515	GEO database	10	10
GSE89377	GEO database	13	35

### Univariate Cox Analysis of Differentially Expressed Genes

The clinical data corresponding to the HCC samples in the TCGA database were downloaded and used for analysis. The information on survival time and survival status in the clinical data was extracted, and the clinical survival data and differential gene expression data were integrated. Through the R “survival” package and default parameters, univariate Cox analysis was performed on the integrated data, and the genes with *p* < 0.05 were retained for subsequent analysis.

### Screening of Core Genes

The upregulated and downregulated genes obtained following differential analyses on the data in TCGA and GEO datasets were intersected with the results of univariate Cox analysis using the Venn diagram software (http://bioinformatics.psb.ugent.be/webtools/Venn/), with the results shown as Venn diagrams. Through the STRING database (https://string-db.org), the protein-protein interaction (PPI) analysis of the two groups of genes was carried out, and the results of the interaction analysis were derived. The degree value of each gene in the PPI analysis results was calculated using Cytoscape software (version 3.8.2; www.cytoscape.org), and the PPI network was constructed (14). The genes with different degree values in the network were represented by different colors. According to PPI analysis results, the genes with degree greater than 10 were defined as core genes (15, 16). Multivariate Cox analysis was used to analyze the core genes. Based on the results, patients with HCC were assigned into a high-risk group and a low-risk group. Survival analysis was then conducted using the “survival” package, and receiver operating characteristic (ROC) curves were plotted using the “survivalROC” package.

### Survival Analysis by the Gene Expression Profiling Interactive Analysis (GEPIA) Database

The GEPIA online database (http://gepia2.cancer-pku.cn/#index) was used to obtain the expression status of core genes in multiple tumors in TCGA. Four upregulated genes in the core genes served as a gene set, and the survival analysis of this gene set in HCC and other tumors was conducted by the GEPIA database.

### Sample Collection

HCC tissues and adjacent normal tissues (3 cm away from the edge of the tumor) were surgically obtained from 72 HCC patients treated at the Tianjin First Central Hospital. The tissue samples were stored at −80°C for later use. None of the patients had received radiotherapy or chemotherapy before the operation. All included patients were pathologically diagnosed with HCC. The tumor node metastasis (TNM) stage was determined according to the American Joint Committee on Cancer's TNM classification, and the patients were followed for up to 3 years.

### Immunohistochemistry

Clinical tissues were fixed in 4% paraformaldehyde for 12 h, dewaxed in xylene, rehydrated in descending series of alcohol (100, 95 and 75% alcohol), and heated in 0.01 M citrate buffer for 15–20 min. After cooling to room temperature, the tissues were washed with PBS, blocked with goat serum and left to stand at room temperature for 20 min. Next, the tissues were immunostained with primary antibodies (Abcam, Cambridge, UK) against CDC20 (ab183479, 1:200), TOP2A (ab52934, 1:200), RRM2 (ab172476, 1:200), UBE2C (ab252940, 1:200) or AOX1 (ab197828, 1:200) at room temperature for 1 h. The tissues were incubated with 30 μL secondary goat anti-rabbit immunoglobulin G (ab6721, 1:2000, Abcam) for 1 h at room temperature. Thereafter, the tissues were treated with streptavidin-peroxidase, allowed to stand at 37°C for 30 min, developed with 3,3′-diaminobenzidine tetrahydrochloride for 5–10 min, counterstained for 2 min, and differentiated with hydrochloric acid-ethanol. The tissues were observed under a microscope after conventional dehydration, clearing and mounting.

### RNA Isolation and Quantitation

Total RNA was extracted from tissues with TRIzol reagents (15596-018, Beijing Solarbio Science & Technology Co., Ltd., Beijing, China), with the concentration determined. The extracted RNA was then reversely transcribed into complementary DNA (cDNA) using the cDNA Reverse Transcription Kit (K1622, Reanta Biotechnology Co., Ltd., Beijing, China). Reverse transcription quantitative polymerase chain reaction (RT-qPCR) was conducted on a fluorescence quantitative PCR instrument (ViiA 7, Daan Gene Co., Ltd., of Sun Yat-sen University, Guangzhou, China). The primers were synthesized by TaKaRa Biotechnology Co., Ltd. (Liaoning China) and are shown in Table 2. Glyceraldehyde-3-phosphate dehydrogenase (GAPDH) served as a loading control and the fold changes were calculated using relative quantification (the 2^−ΔΔCt^ method).

**Table 2:** Sequences for RT-qPCR.

**Gene**	**Sequence**
CDC20	Forward: 5′-TCGCATCTGGAATGTGTGCT-3′
	Reverse: 5′-CCCGGGATGTGTGACCTTTG-3′
TOP2A	Forward: 5′-GTCGCTTTCAGGGTTCTTGAGCC-3′
	Reverse: 5′-TGGCATGTTGATCCAAAGCTCTTGG-3′
RRM2	Forward: 5′-CCAAGGACATTCAGCACTGG-3′
	Reverse: 5′-GAAGCCATAGAAACAGCGGG-3′
UBE2C	Forward: 5′-GCTGCCCAGCCTGTCCTTGT-3′
	Reverse: 5′-CAAAACAAAAATACCACAGCTCA-3′
AOX1	Forward: 5′-ATGCCTGTCTGATTCCCATCT-3′
	Reverse: 5′-CATGACACTTGGCAATCCTCT-3′
GAPDH	Forward: 5′-GAGTCCACTGGCGTCTTCAC-3′
	Reverse: 5′-TTCACACCCATGACGAACAT-3′

## Results

### Differential Analysis of the Data in the TCGA-LIHC Samples

**Figure 1:**
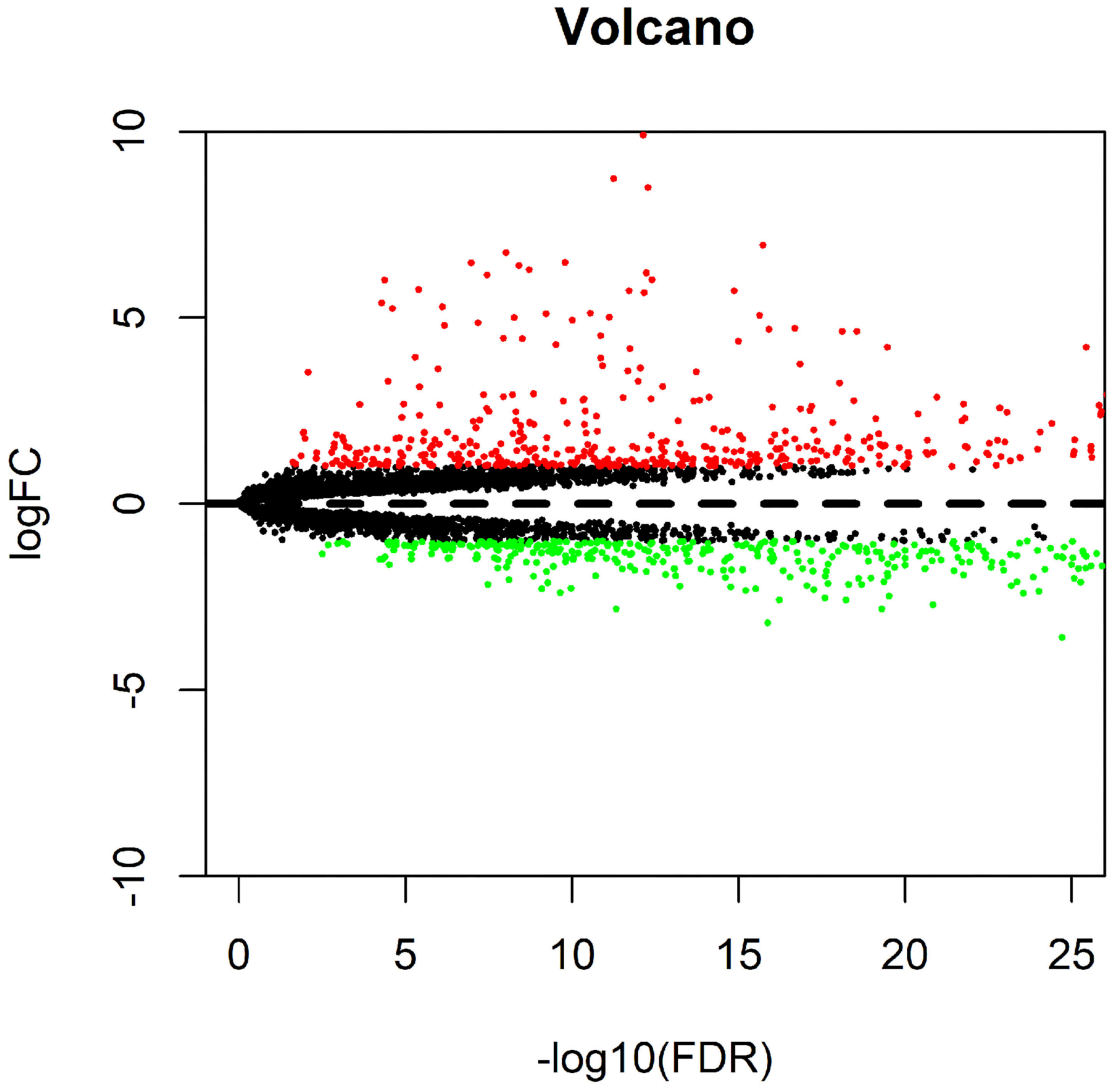
Differential analysis of data in the TCGA-LIHC samples. The volcano map for differential genes is presented, in which the X-axis represents -log10 (FDR), and the Y-axis represents logFC. Each dot in the map represents a gene, where the red dots represent the upregulated genes in HCC, and the green dots represent the downregulated genes in HCC.

Differential analysis of gene expression in TCGA-LIHC samples identified 854 genes that were significantly differentially expressed in HCC samples. Among them, 442 genes were highly expressed and 412 genes were poorly expressed in HCC samples (Figure 1).

### Functional Analysis of the Differentially Expressed Genes in the TCGA-LIHC Samples

**Figure 2:**
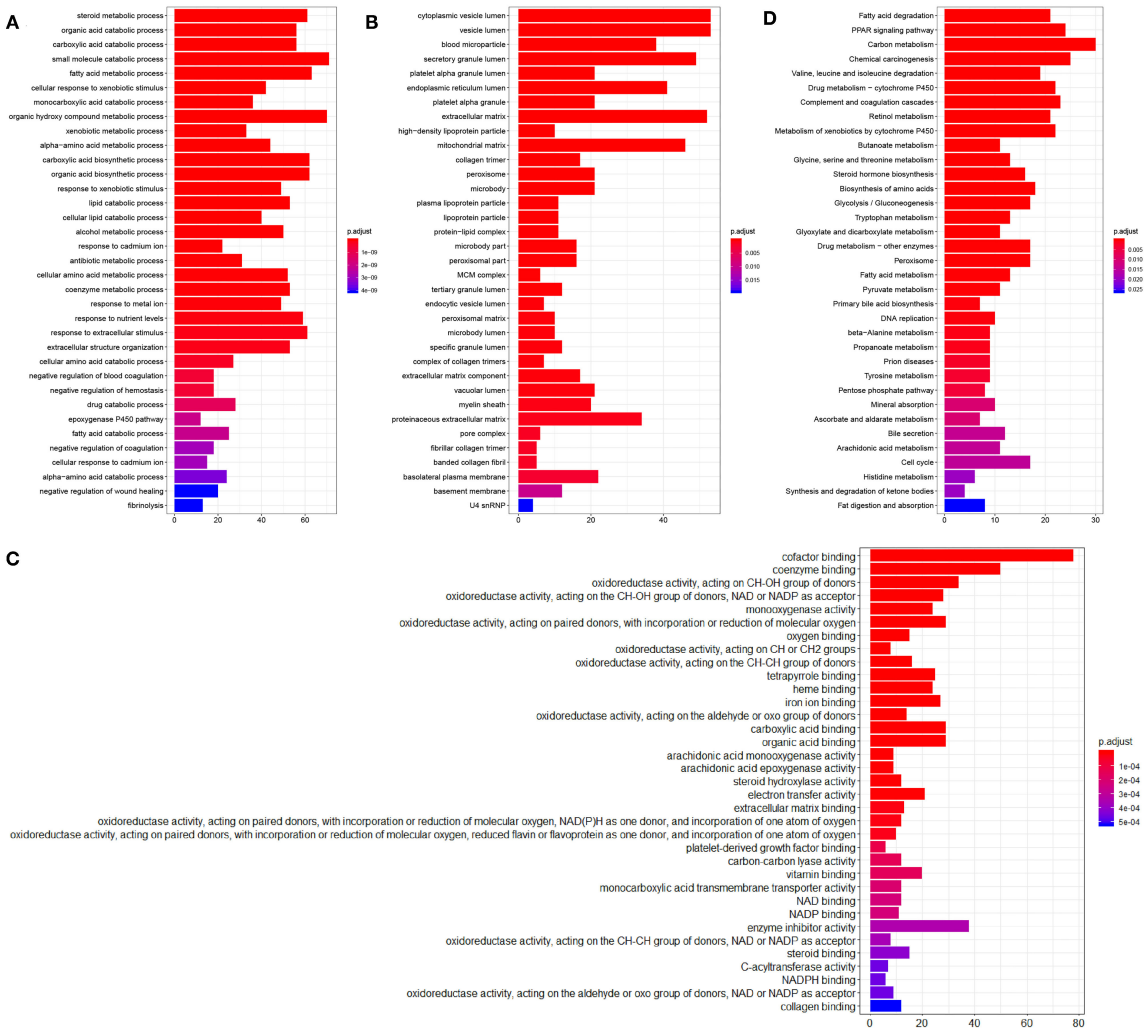
Enrichment analysis of the differentially expressed genes. **(A)**, GO (BP) enrichment analysis of the differentially expressed genes. **(B)**, GO (CC) enrichment analysis of the differentially expressed genes. **(C)**, GO (MF) enrichment analysis of the differentially expressed genes. The X-axis represents the number of genes, the Y-axis represents the functional items, and the histogram on the right is the color scale. **(D)**, KEGG metabolic pathway enrichment analysis of the differentially expressed genes.

A total of 854 differentially expressed genes in HCC samples were then subjected to GO and KEGG enrichment analysis. In biological process (BP) category, these differentially expressed genes were mainly enriched in steroid metabolism and fatty acid metabolism (Figure 2A), while in the cellular component (CC) category, these genes were primarily enriched in vesicle related items (Figure 2B). Molecular function (MF) enrichment results showed that these differentially expressed genes were mainly involved in cofactor binding and other related functions (Figure 2C). Furthermore, KEGG pathway enrichment analysis of these differentially expressed genes revealed the main enrichment in fatty acid degradation, PPAR signaling pathway and other metabolic pathways (Figure 2D). The aforementioned results suggested that the differentially expressed genes in the TCGA-LIHC samples may be involved in the development of HCC.

### Joint Differential Analysis of the Three HCC-Related Gene Expression Datasets in the GEO Database

**Figure 3:**
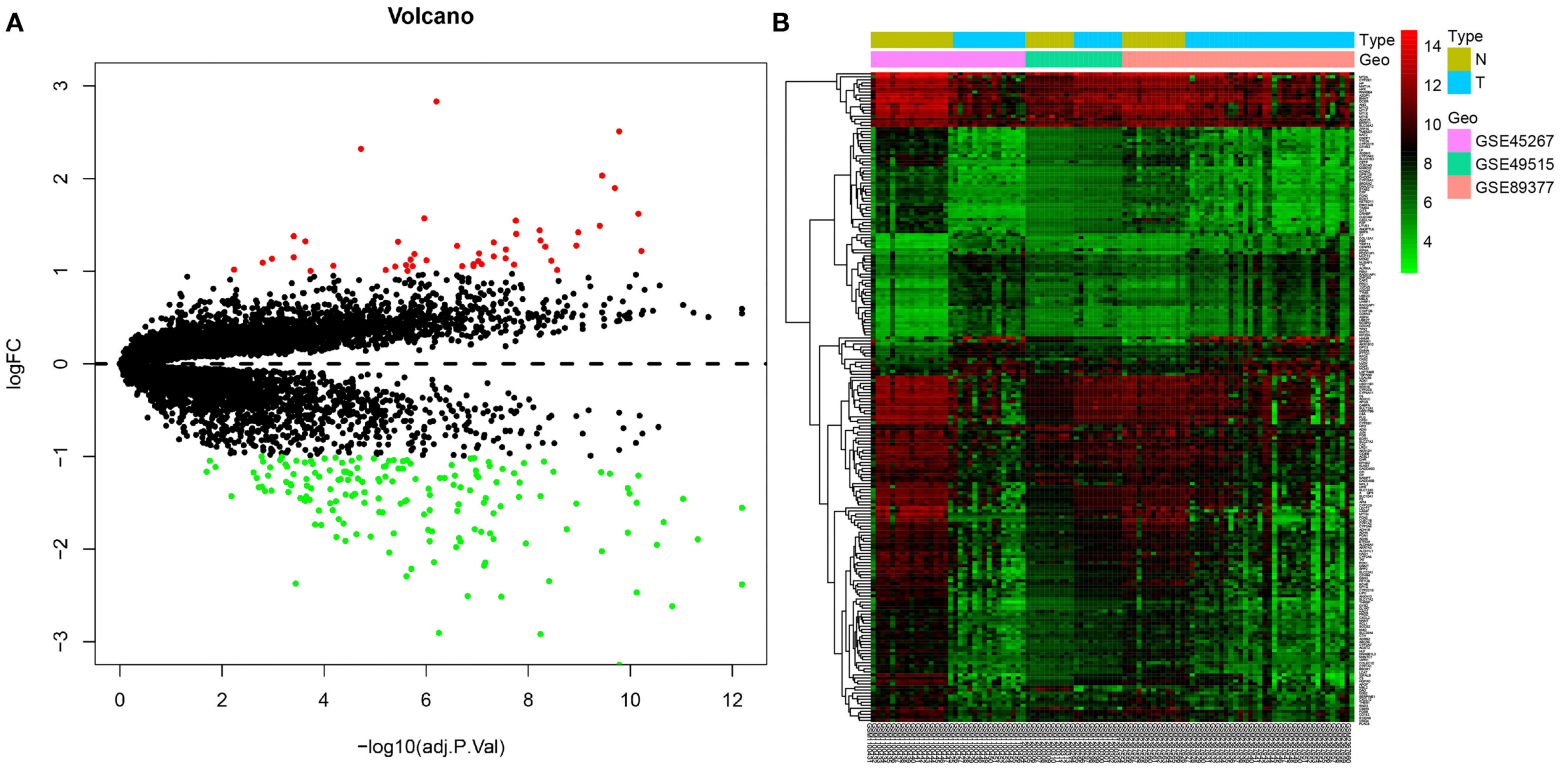
Joint differential analysis of HCC-related expression datasets in the GEO database. **(A)**, A volcano map for differentially expressedf genes in the GSE45267, GSE49515 and GSE89377 datasets downloaded from the GEO database. **(B)**, A heatmap of the differentially expressed genes in the GSE45267, GSE49515 and GSE89377 datasets downloaded from the GEO database. The X-axis represents the sample number, the Y-axis represents the gene name, the left dendrogram represents the gene expression clustering, and the upper right histogram is the color scale.

In order to obtain more accurate gene information related to HCC, three gene expression datasets, namely GSE45267, GSE49515 and GSE89377, were downloaded from the GEO database. Then, the gene expression data contained in the three datasets were analyzed, which yielded 214 genes. Among these, 47 genes were significantly overexpressed in HCC samples, and 167 were significantly underexpressed (Figures 3A,B). The differentially expressed genes from the three datasets obtained from the GEO database were analyzed.

### Joint Screening of the Prognostic-Related Differential Expressed Genes in TCGA and GEO Databases

In order to identify the relationship between the 854 differentially expressed genes in the TCGA-LIHC samples and the prognosis of HCC patients, the clinical data of the corresponding HCC samples were downloaded from the TCGA database, and the survival information of the clinical data was extracted. Univariate Cox analysis of the correlation between these 854 differential genes and survival indicated that the *p* value of 396 genes was less than 0.05 (Supplementary Table 1). The above results suggest that these 396 genes are very likely to affect the prognosis of HCC.

**Figure 4:**
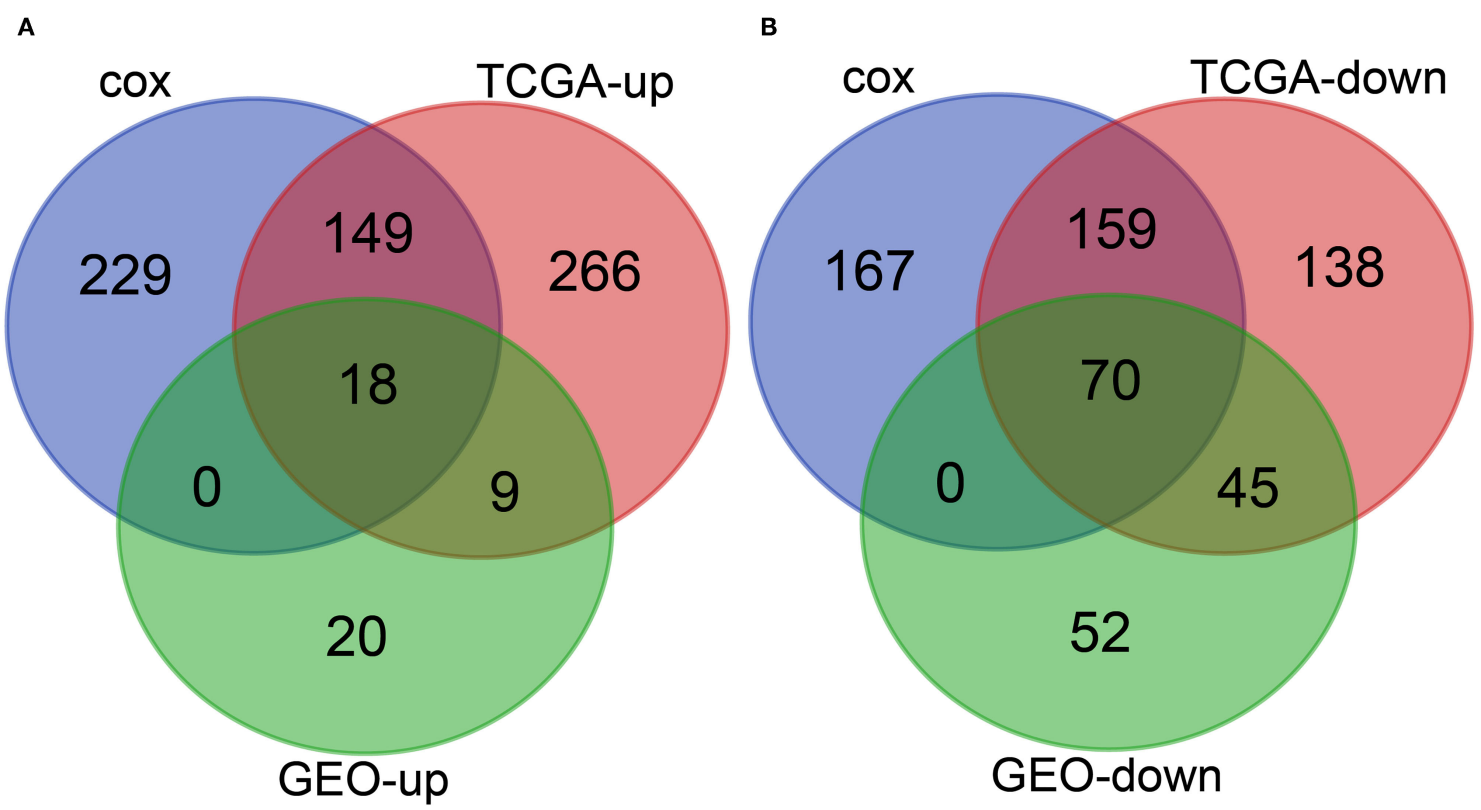
Joint screening of prognosis-related differential genes in the TCGA and GEO databases. **(A)**, Venn diagram of the upregulated genes in HCC samples and the prognosis-related genes. The three circles in the figure respectively represent the upregulated genes in TCGA-LIHC samples, the upregulated genes obtained from the joint differential analysis of the three HCC-related gene expression datasets in the GEO database, and the genes with *p* value less than 0.05 in Cox univariate analysis. The middle part represents the intersection of three groups of data. **(B)**, Venn diagram of the downregulated genes in HCC and prognosis-related genes. The three circles in the figure respectively represent the downregulated genes in TCGA-LIHC samples, the downregulated genes obtained from the joint differential analysis of the three HCC-related gene expression datasets in the GEO database, and the prognosis-related genes obtained from univariate Cox analysis. The middle part represents the intersection of three groups of data.

To further determine the differentially expressed genes related to the prognosis of patients with HCC, we conducted intersection analysis on the upregulated genes in TCGA-LIHC samples, the upregulated genes obtained from the joint differential analysis of the three HCC-related gene expression datasets in the GEO database, and the genes with *p* value < 0.05 in univariate Cox analysis. The combined results revealed 18 upregulated genes related to the prognosis of patients with HCC (Figure 4A). Similarly, intersection analysis was performed on the downregulated genes in TCGA-LIHC samples and joint differential analysis of the three HCC-related gene expression datasets in the GEO database and prognosis-related genes obtained from univariate Cox analysis, which yielded 70 significantly downregulated genes related to prognosis (Figure 4B). The aforementioned analyses indicated a total of 18 upregulated genes and 70 downregulated genes related to the prognosis and survival of patients with HCC.

### Interaction Analysis of 88 Prognosis-Related Differentially Expressed Genes

**Figure 5:**
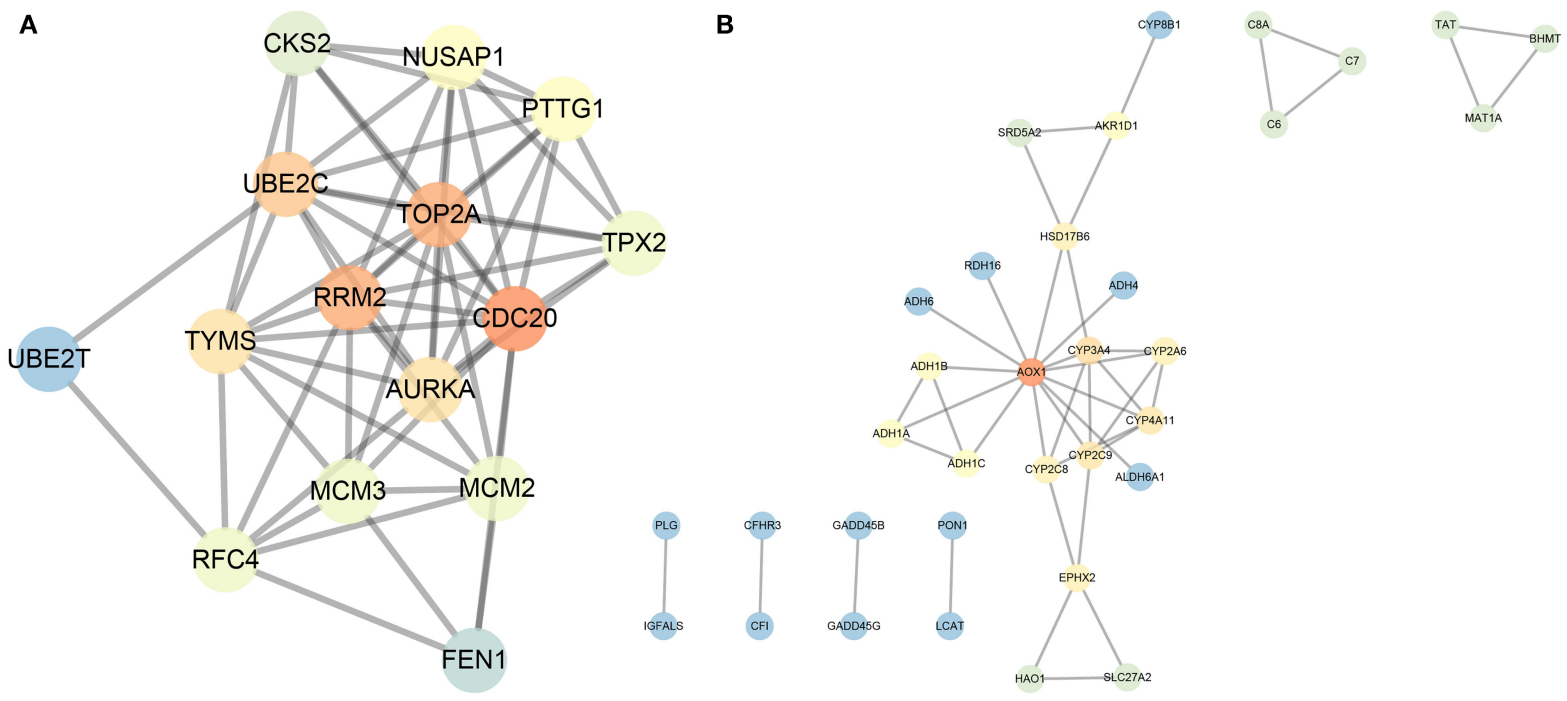
Interaction analysis of the 18 prognosis-related upregulated genes and 70 prognosis-related downregulated genes. **(A)**, Gene interaction network of the 18 prognosis-related upregulated genes. **(B)**, Gene interaction network of the 70 prognosis-related downregulated genes. Each circle represents a gene, and the lines between circles indicate that there is interaction between the genes. The darker the circle, the higher the centrality of the gene in the network, and the greater the degree.

In order to further screen genes closely related to HCC from the obtained 88 genes, we conducted gene interaction analysis between the 18 prognosis-related upregulated genes and 70 prognosis-related downregulated genes, respectively. Through the STRING database, we set the interaction score as 0.9, analyzed the interaction between the above two groups of genes, and counted the degree value of each gene in the network of the two groups of data. We found that in the upregulated gene network, four genes had a degree value greater than 10 (Figures 5A,B), while in the downregulated gene network, only the AOX1 gene had a degree value greater than 10 (Figures 5A,B). The aforementioned data indicate that these five genes are resided at the core of the two networks, and also had significant expression changes in the HCC samples (Table 3), suggesting that these five key genes are not only closely related to the prognosis of HCC, but also play a central role in the progression of HCC.

**Table 3:** Expression difference of core genes and degree value.

**Symbol**	**TCGA-LogFC**	**TCGA-FDR**	**GEO-logFC**	**GEO-FDR**	**Degree**
CDC20	4.063805614	4.41E-30	2.033001075	3.57E-10	12
TOP2A	3.701653345	2.76E-28	1.899209217	2.00E-10	11
RRM2	2.934637946	8.62E-27	1.002622317	0.00018554	11
UBE2C	3.978982289	1.13E-31	1.138571725	2.80E-08	10
AOX1	−1.390530792	1.56E-10	−1.720643658	4.17E-05	13

*CDC20, cell division cycle 20; TOP2A, topoisomerase II alpha; RRM2, ribonucleotide reductase subunit M2; UBE2C, ubiquitin-conjugating enzyme 2C; AOX1, alcohol oxidase 1*.

### Five Key Genes Are Differentially Expressed in a Variety of Tumors in the TCGA Database

**Figure 6:**
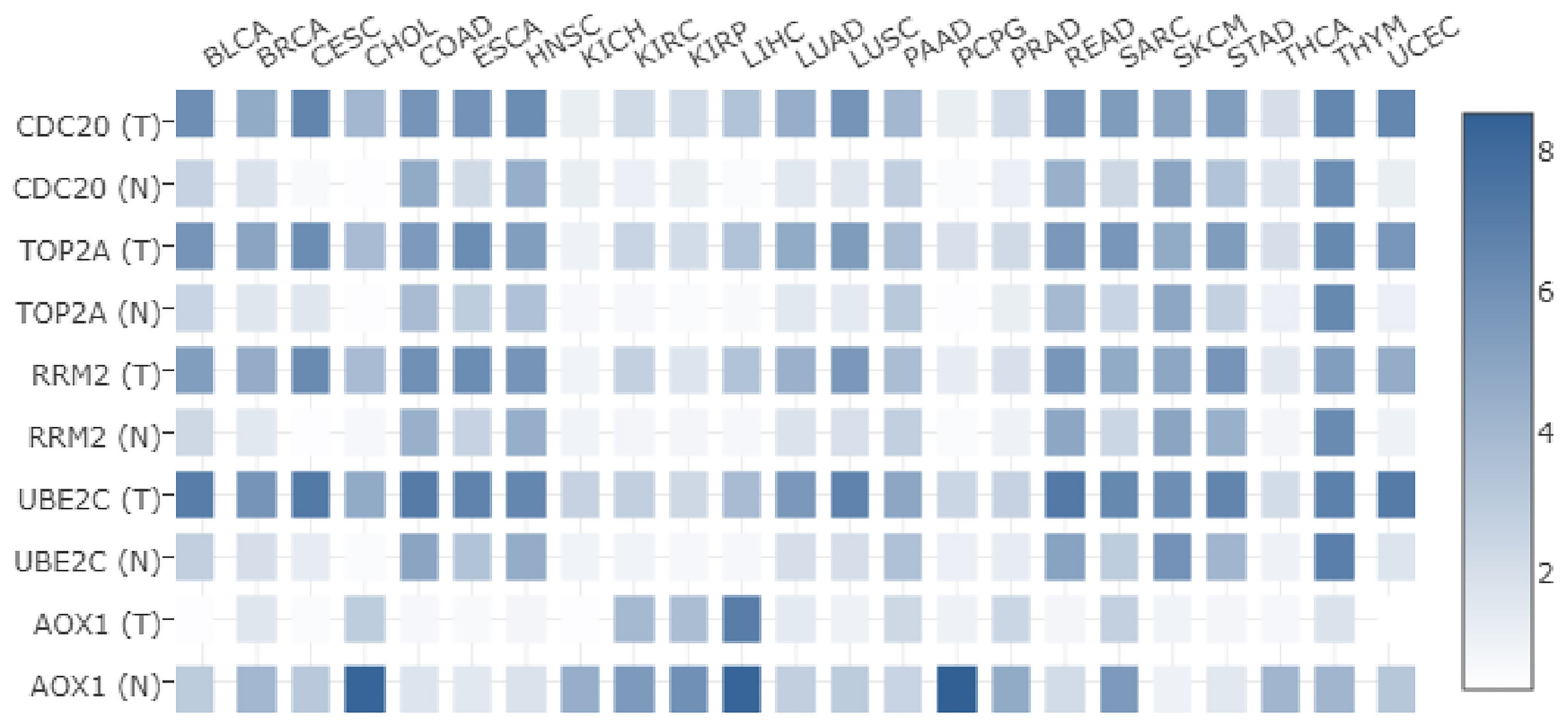
Differential expression of five core genes in many kinds of tumors in TCGA. The X-axis represents the tumor type, the Y-axis represents the gene name. T is the tumor sample, N is the normal sample, and the histogram on the right side is the color scale.

In order to understand the expression of these five key genes in other tumors, their expressions in 23 different kinds of tumors in TCGA were analyzed. As shown in Figure 6, CDC20, TOP2A, RRM2, and UBE2C were highly expressed not only in HCC, but also in breast cancer and other tumors. Meanwhile, the expression of AOX1 was detected to be lower in various tumor samples than in normal samples. Taken together, the five key genes played important regulatory roles in HCC, and furthermore showed significantly differential expression in other tumors. We suppose that this core set of genes may be closely related to the occurrence of a variety of tumors.

### Survival Analysis Reveals That Five Key Genes Are Significantly Related to the Prognosis of Patients With HCC

**Figure 7:**
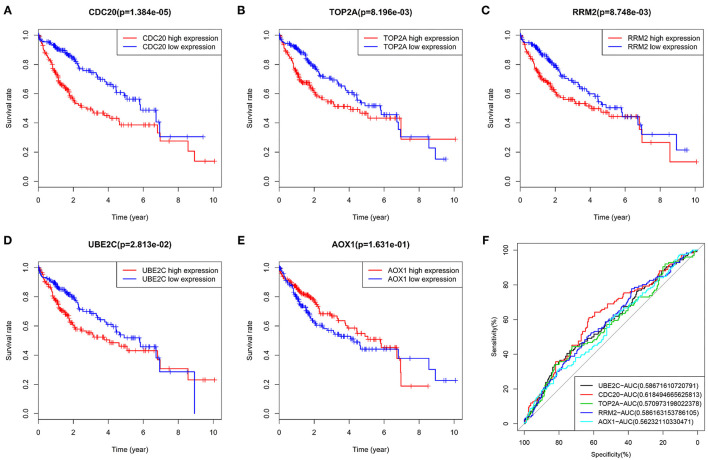
Survival analysis and ROC curve of five core genes in TCGA-LIHC cohort. **(A)**, The survival curve of CDC20 in HCC. **(B)**, The survival curve of TOP2A in HCC. **(C)**, The survival curve of RRM in HCC. **(D)**, The survival curve of UBE2C in HCC. **(E)**, The survival curve of AOX1 in HCC. The X-axis indicates survival time (years), and the Y-axis indicates survival rate. *p* value is shown in the header. Red indicates high gene expression, and blue indicates low gene expression. **(F)**, AUC values of five genes were calculated separately, and ROC curves were drawn. In the figure, the five genes are represented by different colors, and the color code and AUC values are shown at the bottom right.

The five key genes were used for survival analysis in the TCGA-LIHC cohort. The results showed that the four upregulated genes in HCC samples were significantly negatively correlated with the prognosis of patients with HCC (Figures 7A–D). The increased expression of these four genes could significantly reduce the prognosis of patients. On the contrary, low expression of AOX1 in HCC patients was significantly positively correlated with the prognosis of HCC patients (Figure 7E). Thus, the survival of HCC patients with high expression of AOX1 was significantly better than that of patients with low expression of AOX1. At the same time, we made an ROC analysis of these five genes in relation to survival status of patients, and drew an ROC curve. This showed that the AUC values of these five genes all exceeded 0.5, and that the gene with the highest AUC value was CDC20 (Figure 7F), suggesting that it might be a more suitable prognostic marker gene for patients with HCC. Overall, these 5 genes may be key genes affecting the prognosis of HCC.

### Joint Survival Analysis of the Four Prognosis-Related Upregulated Gene Set Shows the Specificity in HCC

**Figure 8:**
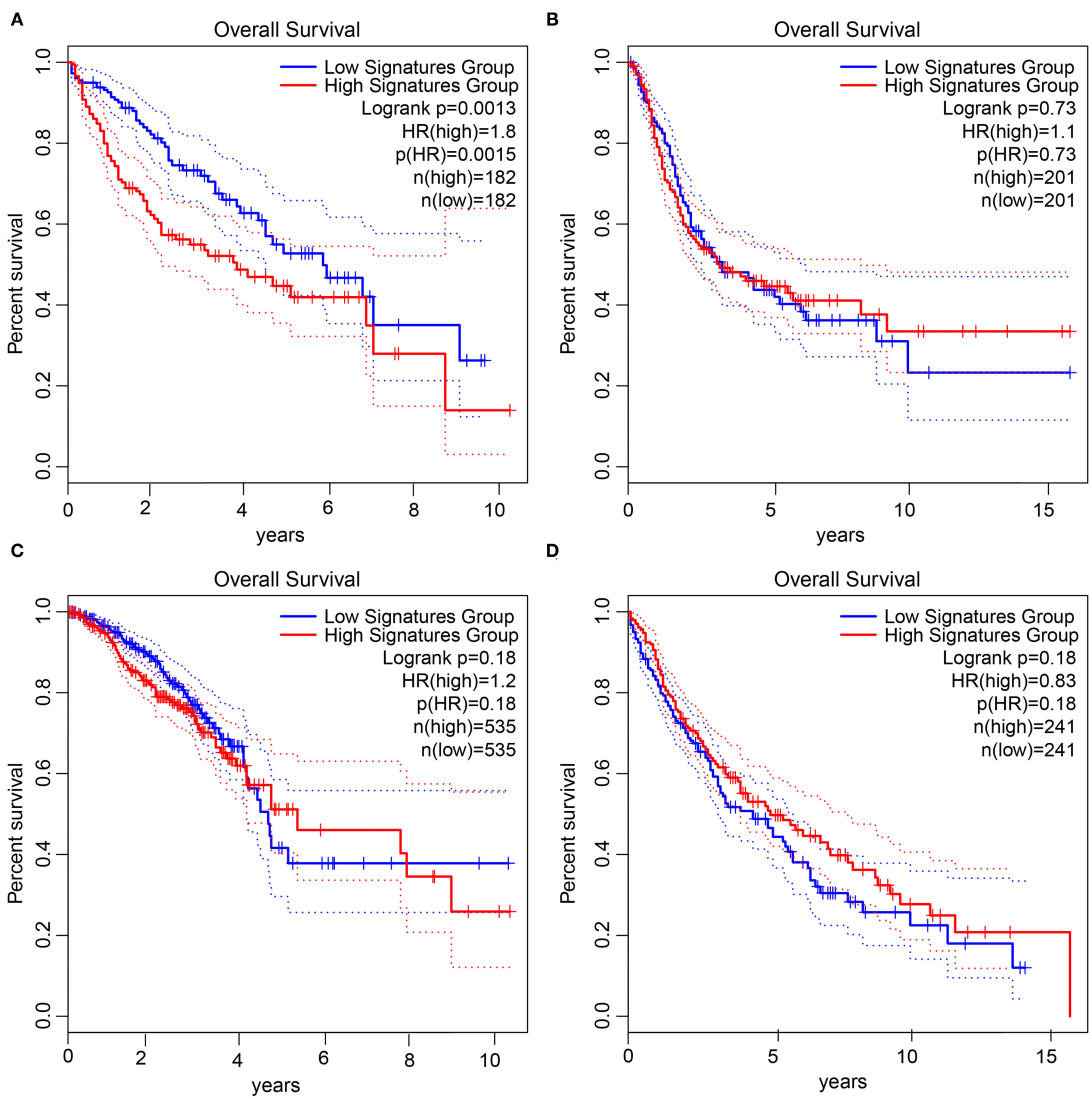
Joint survival analysis of four prognosis-related upregulated gene set in tumors. **(A)**, Survival status in HCC. **(B)**, Survival status in bladder urothelial carcinoma. **(C)**, Survival status in breast invasive carcinoma. **(D)**, Survival status in lung squamous cell carcinoma. The X-axis represents the survival time in months, and the Y-axis represents the survival rate. The upper right corner shows the color code, *p* value, sample information, etc. The red line is the high gene expression group, the blue line is the low gene expression group, and the dotted line is the 95% confidence interval line.

We took the four prognosis-related upregulated genes as a gene set, and used GEPIA database to analyze the relationship between this gene set and the prognosis of HCC. The results showed that the survival time of patients with upregulated genes was significantly lower than that of patients with downregulated genes in HCC (Figure 8A). Meanwhile, we performed a similar survival analysis in patients with bladder urothelial carcinoma, breast invasive carcinoma, and lung squamous cells. This survival analysis (Figures 8B–D) showed that the expression of this gene set was not significantly associated with survival of corresponding patients in these three tumors. Thus, the combination of these four prognosis-related upregulated genes may be closely related to prognosis for HCC.

### Successful Construction of a Prognostic Risk Model Consisting of CDC20, TOP2A, RRM2, UBE2C and AOX1 Genes

**Figure 9:**
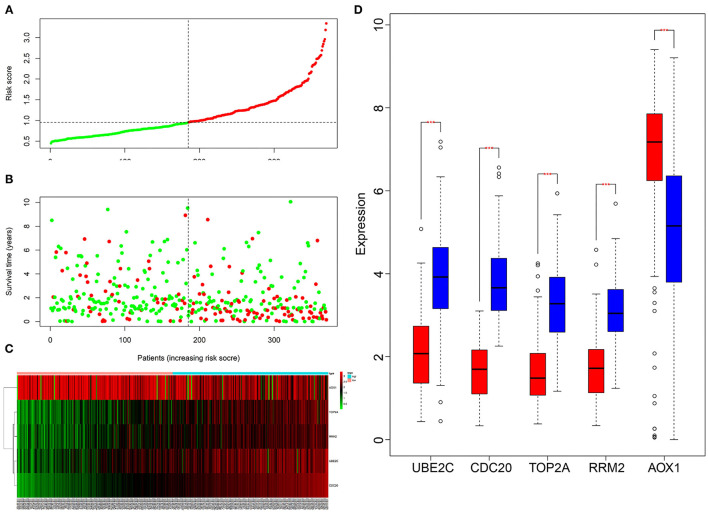
Multivariate Cox analysis of the correlation between five key genes and the prognosis of HCC patients. **(A)**, The patients were assigned into low-risk group and high-risk group. The red X-axis represents the number of patients and the Y-axis represents the risk score. **(B)**, Survival rats of patients, Each dot in the graph represents a patient. The red point represents a patient who had died during follow-up, and the green point represents a surviving patient. The X-axis represents the sample increasing with the risk score, and the Y-axis represents the survival time of the patient. **(C)**, The X-axis represents the sample number, the Y-axis represents the gene name, and the upper right histogram is the color scale. **(D)**, The X-axis shows the gene name, and the Y-axis shows the expression; the red box chart shows the low-risk samples, and the blue box chart shows the high-risk samples (**p < 0.001).

Next, we aimed to elucidate the impact of the five core genes on the survival of HCC patients. Multivariate Cox analysis was performed on the five genes, and the risk value of each gene for each patient was calculated. According to the risk value, the TCGA-LIHC samples were assigned into the high-risk and low-risk groups (Figure 9A). In addition, by analyzing the death status of high-risk and low-risk group, we found that the number of death samples was significantly higher than that of survival samples in the high-risk group (Figure 9B). At the same time, the expression of the five key genes was analyzed in HCC samples, and the expression heatmap and box plot were drawn. As depicted in Figures 9C,D, the expression of CDC20, TOP2A, RRM2 and UBE2C was significantly higher while that of AOX1 was significantly lower in the high-risk group than those in the low-risk group. These results revealed that HCC patients could be classified into a high-risk group and a low-risk group by these five key genes.

### A Prognostic Risk Model Consisting of CDC20, TOP2A, RRM2, UBE2C and AOX1 Genes Accurately Predicts the Prognosis of Patients With HCC

**Figure 10:**
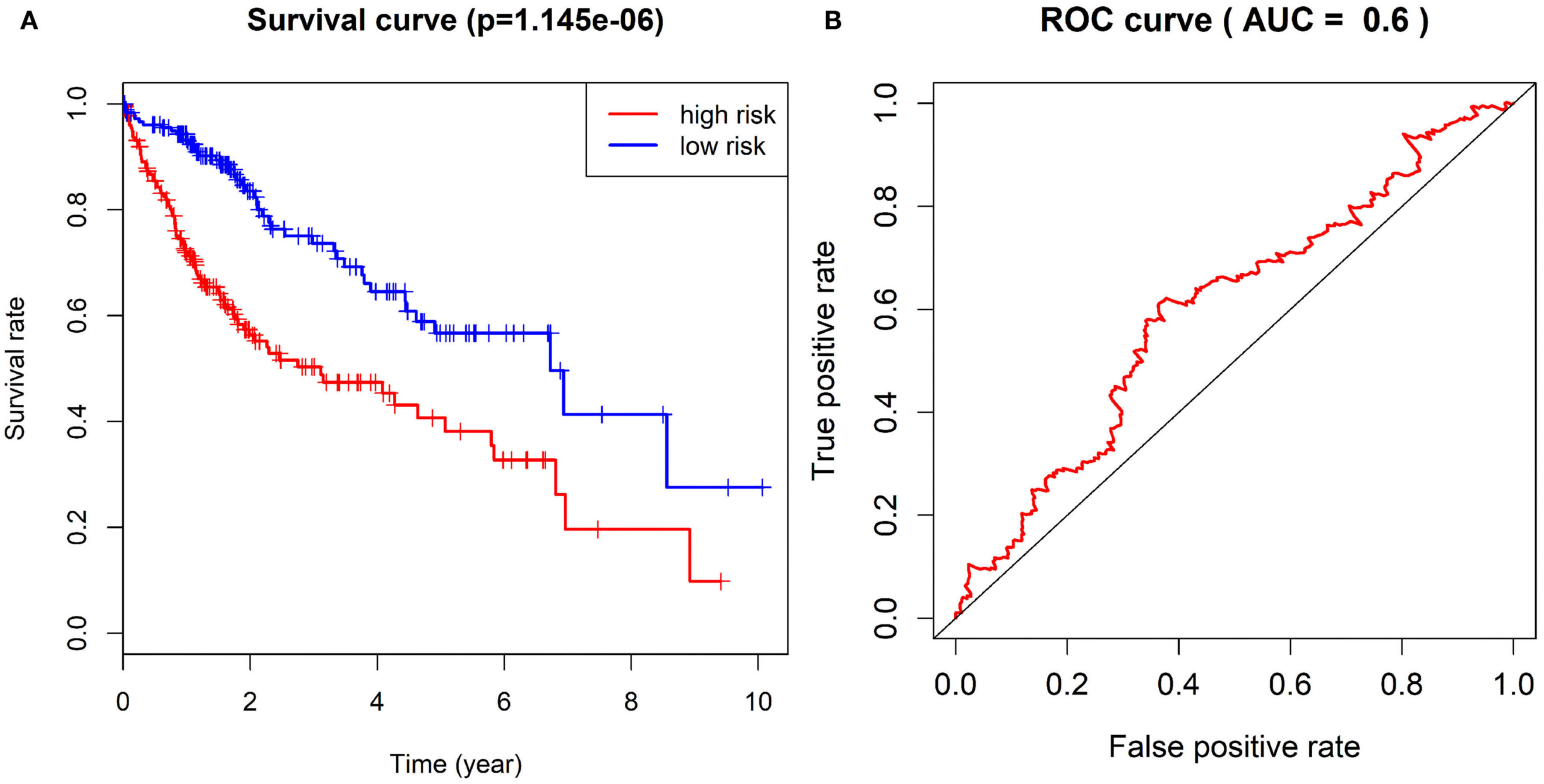
Survival curve of patients with HCC in TCGA-LIHC cohort in the high-risk group and the low-risk group and ROC curve for evaluation of the prognostic risk model. **(A)**, The difference of survival rate between the high-risk group and the low-risk group by Kaplan-Meier survival analysis. **(B)**, The sensitivity and specificity of ROC curves to show risk score in evaluating survival.

According to the expression of CDC20, TOP2A, RRM2, UBE2C and AOX1 and the survival status of patients, we constructed the multivariate Cox analysis model. The HCC patients in TCGA-LIHC cohort were assigned into a high-risk group and a low-risk group, between which the survival status and gene expression of HCC patients significantly differed. Using the univariate Kaplan-Meier survival curve, the survival status of HCC patients in the two groups was analyzed, and the survival curve was constructed. The results showed that the survival rate of HCC patients in the high-risk group was significantly lower than that of the low-risk group (Figure 10A), and furthermore the ROC curve further displayed that the combination of these five genes could be used as a marker gene for survival prediction (AUC = 0.6) (Figure 10B). Overall, these results imply that the prognostic risk model based on 5 key genes can accurately predict the prognosis of patients with HCC.

### Correlation of the Expression of Five Key Genes With the Clinical Characteristics of Patients With HCC

**Figure 11:**
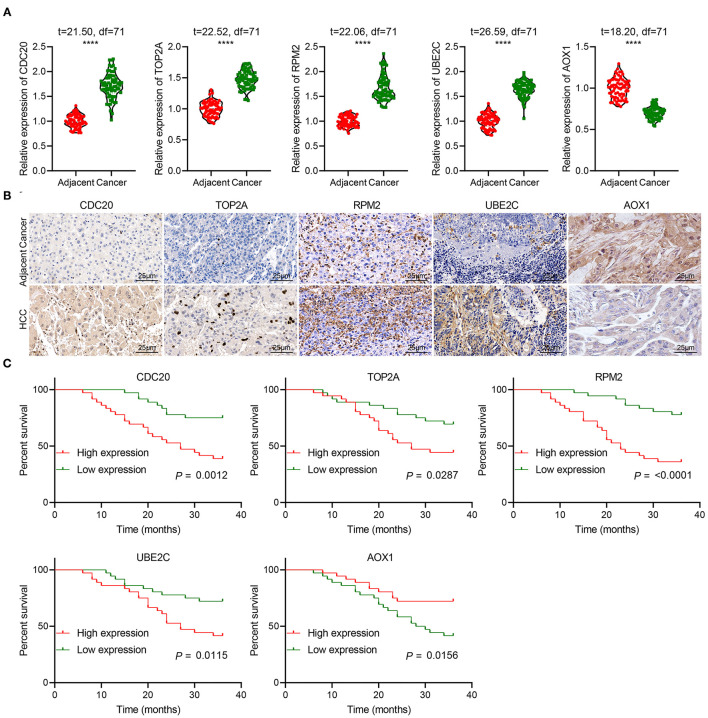
The clinical significance of five key genes in patients with HCC. **(A)**, RT-qPCR detection of CDC20, TOP2A, RRM2, UBE2C and AOX1 expression in the cancer and adjacent normal tissues from 72 HCC patients. **(B)**, Immunohistochemistry analysis of CDC20, TOP2A, RRM2, UBE2C and AOX1 proteins in the cancer and adjacent normal tissues from 72 HCC patients (400 ×). *n* = 10. **(C)**, Kaplan-Meier analysis of the relationship between the expression of 5 key genes and the prognosis of 72 patients with HCC. The X-axis represents survival time (months), and the Y-axis represents survival rate. ****p < 0.0001.

We then focused on identifying the potential clinical significance of five key genes in HCC. RT-qPCR showed that CDC20, TOP2A, RRM2 and UBE2C were significantly highly expressed, while AOX1 was weakly expressed in clinical tissues of HCC patients, which was consistent with the results of our bioinformatics analysis (Figure 11A). Immunohistochemistry results also verified this result (Figure 11B). Kaplan-Meier survival analysis indicated that high expression of CDC20, TOP2A, RRM2 and UBE2C, and poor expression of AOX1 were significantly associated with the poor prognosis of HCC patients (Figure 11C). These lines of evidence provided evidence indicating that these 5 key genes are, taken together, potential independent biomarkers for predicting the prognosis of patients with HCC.

## Discussion

HCC, a malignancy which is featured by a poor prognosis, causes an increasing global health problem because of the generally late diagnosis as well as the inadequacy available therapies (17). HCC is also reported to be inflammation-associated tumor, wherein immunosuppressive microenvironment can induce immune tolerance of HCC and evasion by different mechanisms (18). In the current study, we performed joint analysis of TCGA and GEO databases to explore the prognostic marker genes and immune cells related to the prognosis of HCC. The analysis revealed a suite of five genes, CDC20, TOP2A, RRM2, UBE2C and AOX1, that may be the key genes affecting the prognosis of HCC. The risk assessment model based on the five genes could effectively predict the prognosis of HCC patients with AUC of 0.61. In addition, preferential infiltration of M0 macrophages is a marker predicting poor prognosis of HCC patients.

In the first phase of the study, we performed database-based differential analysis to screen key genes related to HCC, and then conducted a series of analyses, such as univariate Cox analysis, integration analysis and gene interaction analysis. The final results identified CDC20, TOP2A, RRM2, UBE2C and AOX1 as the five core genes in HCC. Among them, CDC20, TOP2A, RRM2 and UBE2C were upregulated in HCC but AOX1 was downregulated. Many previous studies have discovered the implication of CDC20, TOP2A, RRM2 and UBE2C and AOX1 in HCC. For instance, CDC20 was found to a differentially upregulated gene in HCC that was enriched in the “cell cycle” pathway (19). CDC20 and TOP2A are two hub genes that were implicated in the pathological development from cirrhosis to HCC (20). According to the results of TCGA-LIHC dataset-based gene co-expression network analysis conducted by Gu et. al., UBE2C was discovered as one of the hub genes in HCC, which shared a high association with histologic grade in HCC and was a predictor of poor prognosis of the patients (21). In addition, Wei et al. also found that HCC patients with an elevated messenger RNA level of UBE2C had a significantly reduced survival time after diagnosis, suggesting that the overexpressed UBE2C may serve as a potential prognostic biomarker of HCC (22). Inhibition of RRM2 by sorafenib could partially result in the enhanced anticancer activity of sorafenib in HCC cells (23). Importantly, TOP2A, RRM2 and UBE2C were identified previously as hub genes in HCC, such that their expression could affect disease-free survival in HCC (24). Moreover, Ma and colleagues found upregulated CDC20 and TOP2A, as well as downregulated AOX1, in HCC samples (25). Besides, it was reported in previous work that AOX1 displayed a complete loss or reduced expression of AOX1 in HCC (10).

## Conclusion

Based on the results obtained in the present study, we conclude that a suite of five core genes, CDC20, TOP2A, RRM2, UBE2C, and AOX1, can serve as potential markers for prediction of prognosis of HCC. Besides, M0 macrophages have the potential to serve as an independent prognostic marker for HCC. These findings may provide a novel direction for seeking a better understanding of risk factors affecting the prognosis of HCC. Published literature has shown that the pathogenic process of genes can be explored by comparing the correlation between the expression of genes and the abundance of different immune cells (26). High expression of macrophages in the parenchyma can mediate the proliferation, migration, and invasion of tumor cells through a variety of molecular mechanisms, and macrophages infiltration is closely correlated with cancer patient prognosis and therapeutic effects (27, 28). Moreover, studies have reported that M0 macrophages can internalize lung tumor-derived exosomes and differentiate into the M2 phenotype, which is characterized by pro-tumorigenic properties (29, 30). We shall probe further the relationship between the five marker genes and immune cells in a subsequent study. In addition, further research is needed to explore whether the expression of the five key genes is correlated with M0 macrophages and to adjust their expression in M0 macrophages by conducting cell experiments, so as to provide a novel option for the treatment of HCC. Although we have excluded an association with several oncological diseases, a cluster analysis on the expression of these five key genes in many kinds of tumors is also required. Since most HCC patients have pre-existing cirrhosis and other underlying liver disease, which impact prognosis, it would be relevant in future studies to also report liver function in terms of Child-Pugh score (31). Finally, the current results do not take into account the possible prognostic impact of underlying liver disease, which shall be another factor in ongoing investigations of risk stratification for HCC patients on the basis of these genetic and cellular markers.

## Data Availability Statement

The datasets generated for this study can be found in online repositories. The names of the repository/repositories and accession number(s) can be found in the article/Supplementary Material.

## Ethics Statement

The present study was conducted with the approval of the ethics committee of Tianjin First Central Hospital.

## Author Contributions

DT literature search, concept development, writing and editing manuscript, and revision and approval. YY statistical analysis, writing sections of manuscript, and revision and approval. LZ data collection, data analysis, writing and editing manuscript, and revision and approval. JS writing sections of the manuscript and revision and final approval. WJ revised the figures and tables, and revision and approval of manuscript. All authors contributed to the article and approved the submitted version.

## Conflict of Interest

The authors declare that the research was conducted in the absence of any commercial or financial relationships that could be construed as a potential conflict of interest.

## Publisher's Note

All claims expressed in this article are solely those of the authors and do not necessarily represent those of their affiliated organizations, or those of the publisher, the editors and the reviewers. Any product that may be evaluated in this article, or claim that may be made by its manufacturer, is not guaranteed or endorsed by the publisher.
