# Supplementary material for: A Five-Gene-Based Prognostic Signature for Hepatocellular Carcinoma
Source: Front Med (Lausanne). 2021 Sep 8;8:681388. doi: 10.3389/fmed.2021.681388 (PMC8455941; doi:10.3389/fmed.2021.681388)
Supplement: Supplementary file 1 [file Table_1.DOCX]

**Supplementary Table 1** Univariate Cox analysis of the correlation between the 854 differential genes and survival

| Gene | HR | z | *p* value |
| --- | --- | --- | --- |
| G6PD | 1.414558 | 5.539715 | 3.03E-08 |
| KPNA2 | 1.769137 | 5.363166 | 8.18E-08 |
| LPCAT1 | 1.469533 | 4.867825 | 1.13E-06 |
| CDC20 | 1.387671 | 4.834894 | 1.33E-06 |
| SLC1A5 | 1.354142 | 4.743898 | 2.10E-06 |
| TPX2 | 1.474675 | 4.627681 | 3.70E-06 |
| SPP1 | 1.13816 | 4.581732 | 4.61E-06 |
| PON1 | 0.844497 | -4.5493 | 5.38E-06 |
| ACAT1 | 0.661955 | -4.45472 | 8.40E-06 |
| CCNB1 | 1.474748 | 4.446578 | 8.72E-06 |
| FTCD | 0.834718 | -4.40704 | 1.05E-05 |
| CYP2C9 | 0.854081 | -4.39373 | 1.11E-05 |
| ADH4 | 0.882865 | -4.37377 | 1.22E-05 |
| IVD | 0.603139 | -4.35197 | 1.35E-05 |
| LCAT | 0.767203 | -4.34328 | 1.40E-05 |
| ALDH2 | 0.694403 | -4.3432 | 1.40E-05 |
| SPP2 | 0.853147 | -4.31291 | 1.61E-05 |
| ANXA10 | 0.773724 | -4.298 | 1.72E-05 |
| CFHR3 | 0.824884 | -4.28096 | 1.86E-05 |
| STIP1 | 2.081291 | 4.264767 | 2.00E-05 |
| PAGE1 | 1.201215 | 4.264442 | 2.00E-05 |
| MYBL2 | 1.289647 | 4.217932 | 2.47E-05 |
| LDHD | 0.75441 | -4.2047 | 2.61E-05 |
| DNASE1L3 | 0.720899 | -4.1934 | 2.75E-05 |
| AFM | 0.844095 | -4.12281 | 3.74E-05 |
| MCM6 | 1.549751 | 4.095541 | 4.21E-05 |
| SLC2A2 | 0.847518 | -4.01999 | 5.82E-05 |
| PIGU | 1.7939 | 4.010537 | 6.06E-05 |
| CISH | 0.653274 | -3.99594 | 6.44E-05 |
| LMNB2 | 1.590288 | 3.978311 | 6.94E-05 |
| TUBG1 | 1.685178 | 3.96719 | 7.27E-05 |
| SF3B4 | 1.753238 | 3.903512 | 9.48E-05 |
| DUSP10 | 0.67243 | -3.89703 | 9.74E-05 |
| MCM2 | 1.409502 | 3.888875 | 0.000101 |
| BIRC5 | 1.327667 | 3.88124 | 0.000104 |
| ACOT12 | 0.800349 | -3.84932 | 0.000118 |
| SLC16A11 | 0.786503 | -3.84727 | 0.000119 |
| A1BG | 0.818919 | -3.84053 | 0.000123 |
| PTTG1 | 1.344399 | 3.829391 | 0.000128 |
| ZWINT | 1.414275 | 3.821798 | 0.000132 |
| F11 | 0.778848 | -3.81362 | 0.000137 |
| HPX | 0.867974 | -3.80429 | 0.000142 |
| TMEM106C | 1.482048 | 3.787316 | 0.000152 |
| ACADS | 0.66539 | -3.78555 | 0.000153 |
| JPT1 | 1.385362 | 3.767476 | 0.000165 |
| UBE2C | 1.299452 | 3.764186 | 0.000167 |
| HMGCS2 | 0.864149 | -3.73982 | 0.000184 |
| TRNP1 | 1.225068 | 3.738103 | 0.000185 |
| STMN1 | 1.404274 | 3.737975 | 0.000186 |
| SMG5 | 1.688261 | 3.729972 | 0.000192 |
| DTYMK | 1.545716 | 3.725684 | 0.000195 |
| HMGA1 | 1.344685 | 3.714233 | 0.000204 |
| UBE2S | 1.414998 | 3.709444 | 0.000208 |
| FAXDC2 | 0.728573 | -3.70689 | 0.00021 |
| TMEM220 | 0.739831 | -3.6857 | 0.000228 |
| SMIM14 | 0.697055 | -3.6803 | 0.000233 |
| NDRG2 | 0.691115 | -3.64695 | 0.000265 |
| UGP2 | 0.684828 | -3.63811 | 0.000275 |
| HRG | 0.898953 | -3.62021 | 0.000294 |
| GHR | 0.767123 | -3.61699 | 0.000298 |
| LECT2 | 0.863221 | -3.61629 | 0.000299 |
| RRM2 | 1.381579 | 3.607396 | 0.000309 |
| SLC10A1 | 0.881939 | -3.6042 | 0.000313 |
| SLC22A1 | 0.886391 | -3.599 | 0.000319 |
| NT5DC2 | 1.318789 | 3.584174 | 0.000338 |
| ECHS1 | 0.690127 | -3.58104 | 0.000342 |
| LAPTM4B | 1.296288 | 3.558175 | 0.000373 |
| TXNRD1 | 1.334869 | 3.538414 | 0.000403 |
| RGN | 0.816312 | -3.53317 | 0.000411 |
| ACSM2A | 0.837283 | -3.5281 | 0.000419 |
| MSC | 1.175781 | 3.524727 | 0.000424 |
| TOP2A | 1.290601 | 3.516452 | 0.000437 |
| CPS1 | 0.895993 | -3.5117 | 0.000445 |
| GCDH | 0.705145 | -3.4943 | 0.000475 |
| PEX11G | 0.665443 | -3.48208 | 0.000498 |
| RCL1 | 0.703425 | -3.47432 | 0.000512 |
| NDRG1 | 1.28257 | 3.472227 | 0.000516 |
| CCDC137 | 1.574562 | 3.470876 | 0.000519 |
| SLC52A2 | 1.406722 | 3.47023 | 0.00052 |
| TKT | 1.307201 | 3.47011 | 0.00052 |
| SERPING1 | 0.758415 | -3.46605 | 0.000528 |
| DMGDH | 0.806276 | -3.46368 | 0.000533 |
| LMNB1 | 1.376343 | 3.454608 | 0.000551 |
| SERPINF2 | 0.865136 | -3.44616 | 0.000569 |
| CKS2 | 1.392086 | 3.440275 | 0.000581 |
| SLC41A2 | 0.734486 | -3.43458 | 0.000593 |
| GADD45G | 0.814217 | -3.42594 | 0.000613 |
| ADH1B | 0.883459 | -3.41618 | 0.000635 |
| C1S | 0.793201 | -3.41582 | 0.000636 |
| RDH16 | 0.868913 | -3.40801 | 0.000654 |
| ALAS1 | 0.763081 | -3.40531 | 0.000661 |
| PANK1 | 0.676645 | -3.39651 | 0.000683 |
| H2AFZ | 1.486569 | 3.390831 | 0.000697 |
| GLYATL1 | 0.832101 | -3.37556 | 0.000737 |
| KLKB1 | 0.80253 | -3.37117 | 0.000749 |
| SLC27A5 | 0.859376 | -3.36206 | 0.000774 |
| C1RL | 0.704266 | -3.34355 | 0.000827 |
| TAT | 0.901188 | -3.33106 | 0.000865 |
| ALDOA | 1.297802 | 3.327072 | 0.000878 |
| CYP4V2 | 0.729941 | -3.32661 | 0.000879 |
| ADH1A | 0.881124 | -3.3229 | 0.000891 |
| SORD | 0.804188 | -3.32205 | 0.000894 |
| EPS8L3 | 1.226404 | 3.314433 | 0.000918 |
| GABARAPL1 | 0.762243 | -3.31148 | 0.000928 |
| ANG | 0.84008 | -3.30825 | 0.000939 |
| MCM4 | 1.412409 | 3.300892 | 0.000964 |
| C6 | 0.859175 | -3.30069 | 0.000964 |
| SFN | 1.148641 | 3.298832 | 0.000971 |
| UBAP2L | 1.712617 | 3.293484 | 0.00099 |
| RFC4 | 1.490064 | 3.286728 | 0.001014 |
| FMO3 | 0.87615 | -3.27493 | 0.001057 |
| HAO1 | 0.86387 | -3.26812 | 0.001083 |
| FOXO1 | 0.659237 | -3.24368 | 0.00118 |
| SARDH | 0.7931 | -3.23337 | 0.001223 |
| CDC25B | 1.372641 | 3.218757 | 0.001287 |
| AGXT | 0.885151 | -3.21639 | 0.001298 |
| CCT3 | 1.553155 | 3.212894 | 0.001314 |
| ITIH1 | 0.875336 | -3.19925 | 0.001378 |
| MASP1 | 0.765525 | -3.19327 | 0.001407 |
| VPS72 | 1.662345 | 3.187994 | 0.001433 |
| SPRYD4 | 0.648244 | -3.18009 | 0.001472 |
| ASS1 | 0.800692 | -3.17413 | 0.001503 |
| ALDH8A1 | 0.841435 | -3.16847 | 0.001532 |
| PON3 | 0.820664 | -3.16708 | 0.00154 |
| HSD17B13 | 0.901097 | -3.15706 | 0.001594 |
| UBE2T | 1.361804 | 3.152585 | 0.001618 |
| GRHPR | 0.738255 | -3.15217 | 0.001621 |
| CACYBP | 1.607449 | 3.151621 | 0.001624 |
| ILF2 | 1.704445 | 3.147722 | 0.001645 |
| AP5Z1 | 1.717109 | 3.147416 | 0.001647 |
| RNASE4 | 0.745872 | -3.14679 | 0.001651 |
| MT-CYB | 0.746045 | -3.14066 | 0.001686 |
| ADH1C | 0.908018 | -3.13484 | 0.001719 |
| DUSP12 | 1.779098 | 3.119402 | 0.001812 |
| SQSTM1 | 1.316267 | 3.107517 | 0.001887 |
| GYS2 | 0.860297 | -3.10597 | 0.001897 |
| GIT1 | 1.613959 | 3.10036 | 0.001933 |
| PKM | 1.190866 | 3.084576 | 0.002038 |
| FEN1 | 1.400007 | 3.077158 | 0.00209 |
| OGDHL | 0.84073 | -3.07345 | 0.002116 |
| ETS2 | 0.748316 | -3.06255 | 0.002195 |
| CYP7A1 | 0.882512 | -3.04902 | 0.002296 |
| C8B | 0.861126 | -3.04837 | 0.002301 |
| PDK4 | 0.839941 | -3.03872 | 0.002376 |
| SLCO1B1 | 0.867584 | -3.03845 | 0.002378 |
| ABAT | 0.841658 | -3.03564 | 0.0024 |
| ALDOB | 0.909117 | -3.03057 | 0.002441 |
| GNMT | 0.886432 | -3.01811 | 0.002544 |
| PGLYRP2 | 0.879663 | -3.01514 | 0.002569 |
| ME1 | 1.20731 | 2.987503 | 0.002813 |
| HSD17B6 | 0.891255 | -2.98222 | 0.002862 |
| PBLD | 0.822795 | -2.96621 | 0.003015 |
| AKR7A3 | 0.877309 | -2.96445 | 0.003032 |
| RIDA | 0.825718 | -2.96355 | 0.003041 |
| ADK | 0.700574 | -2.95566 | 0.00312 |
| NDRG3 | 1.487843 | 2.954234 | 0.003134 |
| IDNK | 0.710163 | -2.93493 | 0.003336 |
| AKR1D1 | 0.875499 | -2.9332 | 0.003355 |
| GADD45B | 0.807195 | -2.93145 | 0.003374 |
| QDPR | 0.756902 | -2.92406 | 0.003455 |
| BHMT | 0.897205 | -2.91525 | 0.003554 |
| GC | 0.877389 | -2.9121 | 0.00359 |
| SLC22A10 | 0.818733 | -2.91036 | 0.00361 |
| PYGO2 | 1.596802 | 2.910036 | 0.003614 |
| CENPW | 1.294815 | 2.908819 | 0.003628 |
| ETV4 | 1.174678 | 2.891434 | 0.003835 |
| ANO1 | 0.846452 | -2.89046 | 0.003847 |
| DEPDC7 | 0.783291 | -2.88807 | 0.003876 |
| FBP1 | 0.874287 | -2.88425 | 0.003923 |
| HAAO | 0.816761 | -2.87621 | 0.004025 |
| RAP2A | 1.384651 | 2.870765 | 0.004095 |
| SULT2A1 | 0.906426 | -2.86221 | 0.004207 |
| TTYH3 | 1.302446 | 2.856906 | 0.004278 |
| CDK16 | 1.480011 | 2.855494 | 0.004297 |
| CBR4 | 0.712299 | -2.82316 | 0.004755 |
| TRIM16L | 1.194768 | 2.81324 | 0.004905 |
| EFNA4 | 1.391207 | 2.811232 | 0.004935 |
| IRAK1 | 1.387082 | 2.810903 | 0.00494 |
| CAT | 0.795261 | -2.81071 | 0.004943 |
| TTC36 | 0.880408 | -2.80912 | 0.004968 |
| APOA1 | 0.91851 | -2.80873 | 0.004974 |
| AMDHD1 | 0.844141 | -2.80262 | 0.005069 |
| MAGEA3 | 1.135003 | 2.791417 | 0.005248 |
| NR1I2 | 0.828478 | -2.78985 | 0.005273 |
| PCNA | 1.409983 | 2.789602 | 0.005277 |
| MT-ND4L | 0.857017 | -2.78905 | 0.005286 |
| APOC3 | 0.913655 | -2.78498 | 0.005353 |
| MCM5 | 1.388511 | 2.782958 | 0.005387 |
| GNE | 0.792797 | -2.77546 | 0.005512 |
| FGA | 0.895531 | -2.77526 | 0.005516 |
| MT-ND6 | 0.822021 | -2.77107 | 0.005587 |
| MCM3 | 1.347513 | 2.764615 | 0.005699 |
| MCM7 | 1.288405 | 2.762187 | 0.005742 |
| EHHADH | 0.850643 | -2.75795 | 0.005816 |
| H2AFX | 1.300069 | 2.753028 | 0.005905 |
| CDC37L1 | 0.687203 | -2.74882 | 0.005981 |
| GSTZ1 | 0.756617 | -2.74824 | 0.005992 |
| C7 | 0.8762 | -2.74605 | 0.006032 |
| HGFAC | 0.90374 | -2.74416 | 0.006067 |
| CPN2 | 0.874315 | -2.73172 | 0.0063 |
| AGRN | 1.272883 | 2.731565 | 0.006303 |
| TTR | 0.907905 | -2.73076 | 0.006319 |
| CD14 | 0.832136 | -2.72883 | 0.006356 |
| MT-ATP8 | 0.856008 | -2.72069 | 0.006515 |
| FAM189B | 1.491054 | 2.720115 | 0.006526 |
| NQO1 | 1.092361 | 2.716101 | 0.006606 |
| CA9 | 1.122137 | 2.704907 | 0.006832 |
| CES3 | 0.818462 | -2.70024 | 0.006929 |
| C8A | 0.8845 | -2.69545 | 0.007029 |
| EPHX2 | 0.825867 | -2.69366 | 0.007067 |
| VNN2 | 1.170916 | 2.692295 | 0.007096 |
| C1R | 0.818766 | -2.68842 | 0.007179 |
| E2F1 | 1.214118 | 2.680829 | 0.007344 |
| TUBA1B | 1.307201 | 2.678714 | 0.007391 |
| RCAN1 | 0.752232 | -2.67531 | 0.007466 |
| TCF3 | 1.52606 | 2.669421 | 0.007598 |
| LEAP2 | 0.867717 | -2.66123 | 0.007786 |
| S100A11 | 1.174178 | 2.654425 | 0.007944 |
| IL1RN | 0.836338 | -2.64935 | 0.008065 |
| PROZ | 0.854353 | -2.64832 | 0.008089 |
| S100A10 | 1.242083 | 2.646521 | 0.008132 |
| FCN3 | 0.809557 | -2.64646 | 0.008134 |
| SAC3D1 | 1.35526 | 2.645731 | 0.008151 |
| SAMD1 | 1.455459 | 2.638524 | 0.008327 |
| FGB | 0.907015 | -2.63212 | 0.008486 |
| PLG | 0.894934 | -2.62308 | 0.008714 |
| CYP2C8 | 0.908915 | -2.62089 | 0.00877 |
| AKR1B10 | 1.076435 | 2.598096 | 0.009374 |
| ETFDH | 0.765879 | -2.59756 | 0.009389 |
| MT-ND5 | 0.838577 | -2.59529 | 0.009451 |
| CAPG | 1.181509 | 2.592863 | 0.009518 |
| MSRA | 0.744067 | -2.58669 | 0.00969 |
| AURKA | 1.266642 | 2.582109 | 0.00982 |
| SLC38A4 | 0.887497 | -2.58123 | 0.009845 |
| PLVAP | 0.788446 | -2.57899 | 0.009909 |
| XDH | 0.851252 | -2.57842 | 0.009925 |
| LDLR | 0.752916 | -2.5743 | 0.010044 |
| TOMM40L | 1.451339 | 2.573666 | 0.010063 |
| SHMT1 | 0.840793 | -2.57252 | 0.010096 |
| TYMS | 1.240577 | 2.572484 | 0.010097 |
| MT-ATP6 | 0.769559 | -2.57175 | 0.010118 |
| KIAA1522 | 1.323169 | 2.569428 | 0.010187 |
| ALB | 0.909902 | -2.56443 | 0.010335 |
| MAT1A | 0.884009 | -2.56284 | 0.010382 |
| DPYS | 0.895418 | -2.55502 | 0.010618 |
| AZGP1 | 0.890089 | -2.55011 | 0.010769 |
| KDM8 | 0.808205 | -2.54333 | 0.01098 |
| CYP4A11 | 0.895468 | -2.54273 | 0.010999 |
| SERPINA10 | 0.856644 | -2.52429 | 0.011593 |
| SRD5A2 | 0.840133 | -2.52341 | 0.011622 |
| RNASEH2A | 1.294903 | 2.516731 | 0.011845 |
| MOGAT2 | 0.868333 | -2.51085 | 0.012044 |
| PCK2 | 0.857101 | -2.50474 | 0.012254 |
| CYP4F2 | 0.891396 | -2.49845 | 0.012474 |
| GLMP | 1.376434 | 2.495545 | 0.012576 |
| GTPBP2 | 1.418299 | 2.495201 | 0.012589 |
| HOGA1 | 0.816262 | -2.48368 | 0.013003 |
| TMEM184B | 1.403497 | 2.483666 | 0.013004 |
| MUT | 0.789528 | -2.47988 | 0.013143 |
| ANXA2 | 1.257153 | 2.474336 | 0.013348 |
| GBP7 | 0.877106 | -2.47359 | 0.013376 |
| SNRPB | 1.350031 | 2.473079 | 0.013395 |
| PRPF3 | 1.485546 | 2.467748 | 0.013597 |
| IGFALS | 0.880246 | -2.46373 | 0.01375 |
| PPP1R3B | 0.827576 | -2.45842 | 0.013955 |
| APCS | 0.914109 | -2.45504 | 0.014087 |
| GLA | 1.30774 | 2.451532 | 0.014225 |
| MT-ND3 | 0.782869 | -2.44698 | 0.014406 |
| RAMP1 | 0.887083 | -2.43865 | 0.014742 |
| SCAMP3 | 1.393814 | 2.438261 | 0.014758 |
| AR | 0.836983 | -2.4374 | 0.014793 |
| PPP1R1A | 0.888353 | -2.43181 | 0.015023 |
| PSPH | 1.275499 | 2.430122 | 0.015094 |
| PGGHG | 0.832282 | -2.42804 | 0.015181 |
| TEAD2 | 1.22735 | 2.418841 | 0.01557 |
| TM6SF2 | 0.839181 | -2.41685 | 0.015655 |
| PGM1 | 0.782416 | -2.40978 | 0.015962 |
| UROC1 | 0.894655 | -2.40752 | 0.016061 |
| MYO1B | 0.797171 | -2.3994 | 0.016422 |
| PAFAH1B3 | 1.185659 | 2.393737 | 0.016678 |
| CLIC1 | 1.252813 | 2.392386 | 0.016739 |
| CKLF | 1.323176 | 2.390134 | 0.016842 |
| ADAMTSL2 | 0.835424 | -2.38871 | 0.016907 |
| CSRNP1 | 0.756099 | -2.37826 | 0.017395 |
| SAA4 | 0.907715 | -2.37511 | 0.017544 |
| FGG | 0.907498 | -2.37457 | 0.017569 |
| RHOB | 0.827702 | -2.37335 | 0.017627 |
| TMEM54 | 1.162266 | 2.366975 | 0.017934 |
| F9 | 0.915492 | -2.36598 | 0.017982 |
| CYP8B1 | 0.927322 | -2.36504 | 0.018028 |
| CGREF1 | 1.174906 | 2.362929 | 0.018131 |
| ASPDH | 0.892933 | -2.35547 | 0.018499 |
| FOSB | 0.825493 | -2.35066 | 0.01874 |
| JCHAIN | 0.881704 | -2.3443 | 0.019063 |
| MT-ND4 | 0.797333 | -2.3438 | 0.019088 |
| PCK1 | 0.918958 | -2.34045 | 0.01926 |
| APOF | 0.904936 | -2.33991 | 0.019288 |
| CTAG2 | 1.117049 | 2.327207 | 0.019954 |
| BOP1 | 1.213447 | 2.321621 | 0.020253 |
| FAM50A | 1.276438 | 2.315751 | 0.020572 |
| HMOX1 | 1.179367 | 2.313872 | 0.020675 |
| HMGB2 | 1.255959 | 2.311987 | 0.020778 |
| ACLY | 1.388747 | 2.310955 | 0.020835 |
| CKAP4 | 1.27213 | 2.308449 | 0.020974 |
| CYP3A4 | 0.939646 | -2.30581 | 0.021121 |
| SLC46A3 | 0.876305 | -2.30451 | 0.021194 |
| INHBC | 0.888023 | -2.30194 | 0.021338 |
| ALDH6A1 | 0.859472 | -2.29797 | 0.021564 |
| OTC | 0.91136 | -2.29766 | 0.021581 |
| ACADSB | 0.849826 | -2.29546 | 0.021707 |
| BAIAP2L2 | 1.158389 | 2.291593 | 0.021929 |
| CD5L | 0.857999 | -2.29137 | 0.021942 |
| CDKN2C | 1.230536 | 2.284424 | 0.022347 |
| PABPC1 | 1.26472 | 2.276729 | 0.022802 |
| SLC16A2 | 0.850831 | -2.27641 | 0.022822 |
| ACSL1 | 0.878364 | -2.27586 | 0.022854 |
| GLYAT | 0.909369 | -2.27215 | 0.023077 |
| ADH6 | 0.883114 | -2.26244 | 0.02367 |
| TCF19 | 1.212554 | 2.255228 | 0.024119 |
| RIPK4 | 0.768491 | -2.25425 | 0.024181 |
| DNAJC25 | 0.75528 | -2.25107 | 0.024381 |
| ALDH4A1 | 0.822424 | -2.24905 | 0.024509 |
| ALPL | 0.876914 | -2.24607 | 0.024699 |
| SGK1 | 0.829619 | -2.2337 | 0.025503 |
| AOX1 | 0.914435 | -2.21843 | 0.026526 |
| TK1 | 1.189313 | 2.217874 | 0.026563 |
| MTHFD1 | 0.841471 | -2.21699 | 0.026624 |
| CD302 | 0.798573 | -2.2164 | 0.026664 |
| ACACB | 0.796301 | -2.21305 | 0.026894 |
| TKFC | 0.843141 | -2.21244 | 0.026936 |
| SOX4 | 1.172856 | 2.207162 | 0.027303 |
| BBOX1 | 0.870519 | -2.2066 | 0.027342 |
| TMEM45A | 1.149086 | 2.202096 | 0.027659 |
| CLDN4 | 1.108525 | 2.20088 | 0.027744 |
| FETUB | 0.914531 | -2.19802 | 0.027948 |
| PYCR1 | 1.131442 | 2.19685 | 0.028031 |
| C8orf33 | 1.318989 | 2.194024 | 0.028234 |
| CCL20 | 1.099439 | 2.186724 | 0.028763 |
| HSPB8 | 1.128416 | 2.178843 | 0.029343 |
| TSEN54 | 1.366484 | 2.174027 | 0.029703 |
| ZCCHC24 | 0.748169 | -2.17361 | 0.029735 |
| UCHL1 | 1.109273 | 2.158999 | 0.03085 |
| SERPINA4 | 0.912782 | -2.15678 | 0.031023 |
| STEAP3 | 0.86568 | -2.14582 | 0.031888 |
| ACOX2 | 0.866343 | -2.14424 | 0.032014 |
| MMP7 | 1.112166 | 2.14109 | 0.032267 |
| ERRFI1 | 0.861158 | -2.14094 | 0.032279 |
| TP53I3 | 1.200121 | 2.135085 | 0.032754 |
| ATP6V1C1 | 1.33188 | 2.132019 | 0.033005 |
| CLDN15 | 0.868932 | -2.1256 | 0.033537 |
| HMGCL | 0.798737 | -2.11448 | 0.034474 |
| CXCL1 | 1.105355 | 2.113444 | 0.034563 |
| DSN1 | 1.330154 | 2.112034 | 0.034684 |
| CYP4A22 | 0.894525 | -2.10631 | 0.035177 |
| AP1M2 | 1.097953 | 2.104819 | 0.035307 |
| CFI | 0.86435 | -2.08912 | 0.036697 |
| TMEM82 | 0.891621 | -2.08784 | 0.036813 |
| ADAM15 | 1.272948 | 2.087113 | 0.036878 |
| PLP2 | 1.152541 | 2.084417 | 0.037122 |
| HS3ST3B1 | 0.842553 | -2.08125 | 0.037411 |
| APOL6 | 0.807311 | -2.08086 | 0.037446 |
| MYH4 | 0.895022 | -2.0798 | 0.037544 |
| BSG | 1.214182 | 2.075427 | 0.037947 |
| ROBO1 | 1.163025 | 2.069634 | 0.038487 |
| KDELR3 | 1.159867 | 2.066032 | 0.038825 |
| NAT9 | 1.362463 | 2.06331 | 0.039083 |
| S100P | 1.065572 | 2.0599 | 0.039408 |
| C11orf96 | 0.854117 | -2.05906 | 0.039488 |
| CD24 | 1.094428 | 2.056607 | 0.039724 |
| ADM | 1.17855 | 2.056332 | 0.039751 |
| MFSD10 | 1.204523 | 2.051871 | 0.040182 |
| SPARCL1 | 0.884203 | -2.04265 | 0.041087 |
| ATF3 | 0.824551 | -2.0413 | 0.041221 |
| IER2 | 0.773297 | -2.03898 | 0.041452 |
| TLCD1 | 1.216816 | 2.03692 | 0.041658 |
| GPD1 | 0.895373 | -2.03466 | 0.041885 |
| CYP2A6 | 0.946956 | -2.03443 | 0.041909 |
| NUSAP1 | 1.192969 | 2.029038 | 0.042454 |
| COX7A1 | 0.819162 | -2.02581 | 0.042785 |
| UGT2B10 | 0.922769 | -2.02566 | 0.0428 |
| CMBL | 0.891145 | -2.01285 | 0.044131 |
| SLC7A2 | 0.883266 | -2.01083 | 0.044343 |
| DBN1 | 1.169969 | 2.007289 | 0.044719 |
| SCP2 | 0.866135 | -2.00656 | 0.044797 |
| CKS1B | 1.283826 | 2.002755 | 0.045204 |
| RBP7 | 0.853803 | -2.00254 | 0.045226 |
| LAGE3 | 1.212039 | 1.999606 | 0.045543 |
| SLC27A2 | 0.900283 | -1.99865 | 0.045647 |
| MMP14 | 1.153593 | 1.998413 | 0.045672 |
| C12orf75 | 1.129812 | 1.998094 | 0.045706 |
| AGR2 | 1.096694 | 1.99231 | 0.046337 |
| SPAG5 | 1.212566 | 1.987498 | 0.046867 |
| C19orf48 | 1.200298 | 1.987004 | 0.046922 |
| APOA5 | 0.920445 | -1.98163 | 0.04752 |
| FABP4 | 0.889302 | -1.96574 | 0.049329 |
| EHMT2 | 1.326407 | 1.963356 | 0.049605 |
